# The Use of Calomel in the Army

**Published:** 1863-09

**Authors:** 


### THE USE OF CALOMEL IN THE ARMY.

In the last number of the *Circular* we published an order of Surgeon-General Hammond, excluding Calomel and Tartar Emetic from the army medical Supply Table, on the ground that its use has been indiscriminate, and too often unnecessary and mischievous. This interdict has produced, as might have been expected, a great commotion among the medical hosts both in and out of the army. The *anti-mineral-poison* doctors, who have been indulging for years in a hullabaloo against these and like potent remedies simply because, so far as we could understand them, they were taken from the mineral kingdom, are in ecstasies at this seeming compliment to their medical sagacity; while the *old fogies* or conservatives, who took their first lessons in the treatment of diseases long years ago, consider the order as a radical and presumptuous innovation upon custom and science, that the experience of many generations has sanctioned and sanctified. There is another class of practitioners, who care but little for what was done by the fathers, and who respect still less the " herb," the "water," and all other interlopers in the domain of medicine, that think they see prodigious medical progress in the Surgeon-General; and this rescript they look upon as one of its manifestations.

We confess we feel but little concerned for the results of the movement. We have no apprehensions that sick soldiers will suffer from it. The use of Calomel without therapeutical principles or pathological reasons has been too much the fashion among mere routine physicians, and the art of medicine will gain by restricting it. If there are constitutional affections in the army that seem to demand mercury in their treatment, other, and for such purposes better preparations, are still left to the surgeons for such uses. Blue mass, corrosive-sublimate, mercury and chalk powder, iodide of mercury, &c., are all or each available, and there are but few cases in which these will not be found to answer every medicinal indication of a constitutional nature that can be presented. We grant that calomel is often abused, say the conservatives, yet this order is a general impeachment of the professional intelligence or integrity of army doctors, and therefore it is unjust and improper. But it is said that the Surgeon-General has such indubitable facts as to make it clear that it is both just, proper, and humane. Many surgeons may be trusted to use it, without any fear of its being abused, but there are also many who would resort to it from deficient knowledge or from the force of a pernicious habit. Remove the incompetents, it is said. This has indeed been done already to a great extent, and the business is still going on, but to purge the whole medical corps is a work that can scarcely be done while this war shall last. Instead of wasting time in untying the knot it is far better that it be cut at once, and then wait to learn if the process will work kindly.—*Drug. Circular.*

